# Students’ experiences of interprofessional learning by simulating the Swedish concept of coordinated individual care in primary healthcare: a qualitative analysis

**DOI:** 10.1186/s12909-025-07563-3

**Published:** 2025-06-28

**Authors:** Pernilla Alencar Siljehag, Sofie Guldbrand, Helena Sohlman, Marina Taloyan

**Affiliations:** 1 Department of Neurobiology, Care Science and Society, Academic Primary Health Care Centre, Karolinska Institutet, Stockholm, Sweden; 2 Stockholm Gerontology Research Center, Stockholm, Sweden; 3 Child Healthcare Centre, Region Stockholm, Sweden; 4 Hela Kroppen Fysiocenter, Stockholm, Sweden; 5 Academic Primary Health Care, Region Stockholm, Sweden

**Keywords:** Medical education, Interprofessional learning activity, Coordinated individual plan, Interprofessional competence

## Abstract

**Background:**

The number of students from medical study programs undertaking clinical education and training in primary healthcare is increasing in Sweden. At the same time, the number of patients with complex needs and multimorbidity is also increasing, calling for interprofessional collaboration of care.

**Purpose:**

The study’s purpose was to describe students’ experiences of interprofessional learning (IPL) from a person-centred perspective in a clinical primary care context using an authentic patient case and simulating a coordinated individual plan (CIP) meeting. The research questions were: (1) Do students perceive the CIP meeting as a relevant IPL activity within clinical training? and (2) What is the novelty of using the CIP meeting with regards to the ICCAS (Interprofessional Collaborative Competency Attainment Survey) - self-assessment scale of IPL competences?

**Method and material:**

The current study is based on a qualitative evaluation of 12 learning activities carried out over a one-year period. Activities were performed as simulation-based CIP meetings within clinical education, and interprofessional training under supervision. In total, 87 students from 13 different medical programs participated in the activities. Qualitative content analysis was used.

**Results:**

The identified theme was *Interprofessional collaborations without hierarchy within a patient-centred setting*, and consisted of five categories: (1) patients in center; (2) one’s own professional identity; (3) professional identity of others; (4) teamwork and collaboration; and (5) non-hierarchical positioning.

**Conclusions:**

A novel finding in this study was the students’ experience of creating new knowledge about their own professional identity and non-hierarchical working practices. Additionally, CIP meetings were perceived as a relevant IPL activity in the clinical training of the students.

**Supplementary Information:**

The online version contains supplementary material available at 10.1186/s12909-025-07563-3.

## Introduction

All higher education in the healthcare professions today focuses on interprofessional learning (IPL) as a concrete goal during clinical education in Sweden. Students are naturally confronted with interprofessional care situations during clinical education [1]. More detailed guidelines on how or to what extent this should be included in training are often lacking [2]. The primary care supervisors in Region Stockholm have a minimum requirement for pedagogic competence for their assignment for supervising, but this does not include a more deeply rooted knowledge of what interprofessional collaboration, learning and education entail. Although clinical reality naturally offers a great diversity of interprofessional meetings and potential pedagogic situations, in practice it is often coincidence that determines the opportunities students are exposed to during their work-based education [3]. To achieve the WHO’s 2010 goal of a future healthcare staff that is prepared and competent for concrete interprofessional collaboration, planned learning activities are needed where a student’s full learning potential can be utilized, and where all participants benefit from reflection and feedback on the set learning goals. It is important to create opportunities where students can use their knowledge in critical analysis and evidence-based practice to reflect on how interprofessional collaboration is implemented in care practice and in routine procedures [1].

The challenges include creating patient cases and scenarios that challenge all the professions in the learning activity, as well as providing pedagogical competence for supervision, with both a facilitating and reflective function [4]. To enable healthcare to sharpen its ability to utilize patient and staff competence in unique, complex situations, effective pedagogical support is required during clinical training.

Structured IPL activities with competent pedagogic support, based on authentic routine procedures found in all types of care, can help develop interprofessional competencies in future healthcare professionals [1].

Person-centred care increases the opportunities for cost-effectiveness, patient safety, and patient satisfaction [5–7], and is conceptually close to the conditions for interprofessional collaboration. Care processes where both interprofessional and person-centred perspectives are emphasized, highlight opportunities whereby these two methods can reinforce each other [8]. Interprofessional collaboration has been raised as a priority goal for education, development, and research to ensure good future care, not least in the primary setting [9].

It has been seen that interprofessional training in clinical education can improve students’ skills regarding collaboration, communication, person-centred perspectives, conflict management, teamwork, and roles and responsibilities [10, 11]. Students’ attitudes to collaboration and teamwork also become more positive after clinical training together [12], decreasing the risks of professional centring and harmful stereotyping. Students are often pleasantly surprised when their stereotypes are broken and are positive about collaboration between professions [13]. Interprofessional education has the potential to change the stereotypical perception between healthcare professions and contributes to students’ understanding of the need for person-centred care and integrated care teams [14].

Physical therapy, nursing and social work students stated that they have developed their clinical thinking through interprofessional training regarding care planning before discharge from the hospital [15]. Their awareness of involving the patient in discharge planning was also raised. Simulation exercises involving patients and interprofessional care planning before discharge can improve future effective interprofessional collaboration in practice [15].

The International Nursing Association for Clinical Simulation and Learning set an evidence-based standard for simulation training describing critical thinking, problem solving, clinical reasoning, clinical assessment, and the transfer of theoretical knowledge in practical care work as the strengths of the method [4]. One advantage of using simulations with standardized patients (a person playing a role of patient) is the opportunities it gives students to practice how to set patients at the centre of interprofessional collaboration [16]. The method has already been used successfully to simulate patients’ being discharged from inpatient care to separate residences in the USA [15]. However, there are still no documented experiences of similar training in a Swedish context.

This study aimed to describe students’ experiences of interprofessional learning with a person-centred approach in a clinical primary care context, using an authentic patient case and simulating a CIP meeting.

### Clinical context and relevance of CIP for interprofessional learning

The Academic Healthcare Centres (AHCC) in Region Stockholm have worked systematically with interprofessional training and guidance for students in clinical education. Good experiences have been gathered from several authentic activities for students from all primary care professions, a small part of which has been described scientifically [17, 18]. Learning activities need to be relevant and train students for authentic care situations. Thus, learning activities need to reflect the reality of complex healthcare and the requirements of patient safety and effective care [19]. The ICCAS self-assessment scale was designed to assess the change in interprofessional collaboration-related competencies in healthcare students and practicing clinicians before and after IPE training interventions [20]. The older population with complex care is growing group of patients in Swedish primary care, however new pedagogic models that focus on challenges for the care and for collaboration across professions and caregiver organizations are still lacking.

A prerequisite for future preparedness is that students learn from, about, and with each other in an interprofessional team because of future care needs requirements. Thus, we chose to use Coordinated Individual Plan (CIP) meetings as the basis for an interprofessional learning activity, as CIP is a standardized tool for person-centred interprofessional collaboration in Swedish primary care.

Since 2018, patients with complex needs discharged from hospitals are offered a CIP meeting according to a law in Sweden regarding collaboration between inpatient and outpatient healthcare. CIP is offered to patients who have support from both social service in the municipality and healthcare provided by the Region, and who need a coordinated plan [21].

The task of coordinating and planning interventions from municipalities and regions when older patients are discharged from hospital lies with primary care. For older patients being cared for at home in the Stockholm, the permanent care contact (often a nurse or district nurse) keeps in touch with the nurse at the hospital and the social worker at the municipalities via a common digital communication platform, Lifecare [22]. All members of this collaboration produce a CIP after consent from every single patient. Many professions are often involved in this plan at the primary healthcare centre. Even relatives of the patient can participate or participate instead of the patient. The outcome of the collaboration is a protocol that states the overall background of the patient and a detailed plan, with sub-goals, and the specific responsibilities for each activity/care provider. CIP meeting is based on the patient’s wishes and needs, and proposed solutions are discussed between the patient and the represented professional. Meeting goals must always be followed up with a further meeting, unless there is no explicit need.

IPL activities in clinical education at Hässelby APHCC were based on student-led CIP meetings discussing authentic and anonymized cases of patients who needed care at home after discharge from hospital. The goals of the activities were: (a) to pilot a model for IPL with a focus on the concrete needs of multimorbid older adults for effective collaboration between different actors, both on educational and professional levels; (b) to increase students’ knowledge of other professions’ roles, functions, and responsibilities; (c) to confirm students in their professional roles and strengthen their professional identity using authentic pedagogic opportunities in a real patient case and role simulation; (d) to train students in a person/family-centred manner by creating CIP during simulated meetings; and finally (e) to stimulate integration and interaction between theory and practice through structured preparedness and feedback to IPL goals using ICCAS [20].

The goal of the current study was to describe students’ experiences and perspectives on CIP as an IPL activity, and to discuss the themes identified with the six ICCAS competencies. The research questions of this study were: (1) Do students perceive the CIP meeting as a relevant IPL activity within clinical training? And (2) What is the novelty of using the CIP meeting with regards to the ICCAS competencies?

## Study design

### Method

#### ICCAS in the current study

The current study is based on the qualitative evaluation of IPL by students discussing dimensions from the ICCAS self-assessment scale, as a retrospective pre- and after assessment using a simulated CIP meeting as the basis [20]. This study used the version of ICCAS adapted from Archibald, MacDonald and colleagues [23], which includes the following six dimensions (competences): (a) *communication* with instructions: promote effective communication among members of an interprofessional (IP) team; actively listen to IP team members’ ideas and concerns; express ideas and concerns without being judgmental; provide constructive feedback to IP members; and express ideas and concerns in a clear, concise manner; (b) *collaboration* with instructions: seek out IP team members to address issues; work effectively with IP team members to enhance care; learn with, from, and about IP team members to enhance care; (c) *roles and responsibilities* with instructions: identify and describe one’s abilities and contributions to the IP team; be accountable for one’s contributions to the IP team; understand the abilities and contributions of IP team members; recognize how others’ skills and knowledge complement and overlap one’s own; (d) *collaborative patient/family-centred approach* with instructions: use an IP team approach with the patient to assess the health situation; use an IP team approach with the patient to provide whole person care; include the patient/family in decision-making; (e) *conflict management/resolution* with instruction: actively listen to and take into account the perspectives of IP team members; respectfully address team conflict; (f) *team functioning* with instructions: develop an effective care plan with IP team members; negotiate responsibilities within overlapping scopes of practice.

#### Design of interprofessional learning activity

The data used in the current study are based on qualitative evaluations of the 12 IPL activities over one year. The activities were simulation-based CIP meetings within clinical training under supervision, facilitated by preceptors and adjunct clinical teachers reading a case, followed by interprofessional discussion as *a role-playing game* where respective students took their professional roles. One of the academic clinical teachers had the role of patient and possible relative, based on authentic patient cases, without any personal information on the real patient. In that way, students created concrete experiences through experimental learning [24–26].

Retrospective assessment is supported by previous research that shows that this method reduced the risk of students overestimating their ability before the activity by changing their frame of reference during the activity − the so-called response bias [27]. When the IPL activities were completed, ICCAS was used as a group discussion tool. Individual written evaluations were performed after each IPL activity, and included two questions: (a) What new insights do you take with you from the activity, and (b) how do these connect with your previous knowledge? These qualitative questions were included to evaluate the learning activity, along with student feedback, and were grounds for further improvement of learning during students’ clinical placements. These questions had been used at Hässelby APHCC for many years as a part of students’ feedback during their clinical training and were thus validated for supervisors. All authors performed qualitative content analysis, first individually, and then together in discussion [28].

#### Study population

A total of 87 students from different medical programs attended 12 learning activities during their clinical placements between September 2018 and December 2019 (see Supplementary Table 1 for medical programs and clinical units). Each session included between four and 11 participants. Students from 13 medical programs participated in the IPL activities, including 26 nurses, 16 medical doctors, 13 social workers, nine physiotherapists, and eight district nurses. Students from other programs included dieticians, medical secretaries, occupational therapists, assistant nurses, chiropractors and naprapathy, and paediatric nurses. Evaluations were anonymous, and the results are presented at the group level.

## Results

### Study population

A total of 87 students participated in 12 IPL activities at Hässelby APHCC for one year. The number of students, units they were placed during clinical placements and medical programs differed between activities. However, simulated CIP IPL activity was performed if at least four professions were involved (see Table 1). We identified one theme and five categories with subcategories, respectively, in the qualitative content analysis (see Supplementary Table 1).

The overall theme was identified as a result of the content analysis − *Interprofessional collaboration without hierarchy with patients in center* − constituted by five categories: patient in center, one’s own professional identity, professional identity of others, teamwork and collaboration, and finally, non-hierarchical positioning. Categories are illustrated by quotations from the free text evaluations of the CIP as an IPL activity.

In general, students evaluated simulated CIP meetings using the authentic patient cases good, with credible cases that felt real and that enabled the practice of their own role. Some descriptions are illustrated here:

“… good experience that you take with you in later professional life”; that the activity leads to “… see with different glasses”, and that they gained more insights into and experiences of CIP: “… gained more insight and experience regarding CIP, and it was good to use the case.

### Patient in center

Patient-centred care was emphasized clearly both as a separate statement in free text and as a final and essential purpose. Patients are in the centre and should not be forgotten, as the main goal of all professions is to be the patient’s advocate. One of the students concluded that a new insight for her was patient focus, as the following illustrates:

I learn how to be the patient’s advocate first and foremost.

To make clear a final and important purpose of the care – to set the patient at centre − was pointed out, even in discussions regarding the understanding of other professions’ roles and priorities. The quote below illustrates that whatever is done and understood during the discussions, the main goal and purpose of the meeting was not to forget patient:

I learn what other professions’ responsibility areas are and get a deeper understanding of their priorities. Which is not to forget the patient and his/her wishes!

And:

It is important to negotiate diplomatically with the patient and not have any prejudices regarding other’s perceptions and tasks.

The students experienced that all professions could complement each other for the best possible care of the patient. The patient is at the centre, and you can see patients’ health issues from various perspectives.

### One’s own professional identity

Several comments and conclusions in the material show that the evaluation of the activity was judged with a focus on one’s own profession. Students discussed that it is important to represent their own profession and to practice that presentation. One student expressed it in following way:

I got greater knowledge regarding the importance of representing my profession and at the same time, as a nurse, having responsibility to document and notice other professions’ tasks.

The best experiences were the opportunity to practice their own professional roles and to represent the role, as expressed below:

I could practice my own ability to represent the nurse’s role.

Important to briefly and clearly describe my own professional role and how I can contribute.

To participate in a CIP meeting based on an authentic patient case with other professions raised thoughts and new insights regarding their own professional identities. One student described the experience of participation in the IPL activity in following way:

My experience is that it was very educational for me as a future nurse and is something that we will benefit from in our future careers. To have a case that feels real, and to practice our own roles in that scenario.

The CIP meetings enabled the students to be in control of their role and clearly describe to other professionals how they can contribute. For all these reasons, CIP meetings clarified the need for practice to enable further development of the students’ own professional identity, expressed by one of the students as follows:

“… [it] clarified for me what I need to practice, and what I need to read about”.

### Professional identity of others

The knowledge about how other professions work and their roles in practice is needed. What kind of knowledge do other professionals have? The learning activity, with many professions involved, contributed to understanding other’s roles and the distribution of their responsibilities. By participating in CIP learning activities and discussions with other professions, students got a deeper understanding of different professions and their priorities. Listening to how other professions work, and their points of view in discussion of the same patient case and listening to their perspectives contributed to new knowledge about other professions. Quotations below illustrate this clearly:

I learned a lot about other professions and their roles in practice.

To understand other professions’ insights and what they think is important.

### Teamwork and collaboration

Interprofessional collaboration is important. It gives a holistic picture of patients’ needs and emphasizes the help from team members to find solutions, as the quotation below illustrates:

“… to handle certain situations with the help of others in a team” and “you find the solution with everyone. You get a holistic picture”.

I got a picture of how to handle some situations with help from others in the team.

“…it was good to have everyone’s points of view from different perspectives and professions, and that solutions are reached with everyone. You got an overall picture of the whole thing”.

The summarizing of all the discussions and solutions ended in the main goal − to give the best possible care for the patient, and to reach this goal, teamwork is important:

How to complement each other’s professions for the best care possible for the patient.

“… teamwork is an important key part of how you can help a patient in the right way”.

Team-based work is challenging. However, it contributed to awareness of own role and what to focus on according to students’ perceptions:

It is challenging with team-based work, but it is extremely good. It is good to be aware of one’s own role, so you know what to focus on.

### Non-hierarchical positioning

IPL activity showed that all professions are needed, according to participants in the activity. Without non-hierarchical teamwork, not everyone around the patient can be a part of the interprofessional collaboration. This might be because of stereotypes or working in silos. To show respect and not criticize each other, instead of creating one’s professional silo, can only be better for patients, as illustrated here:

The importance of all professions. Everyone is needed.

## Discussion

Students’ perceptions of simulated CIP meetings for authentic patient cases were, in general, positive, and they believed that it was relevant training activity for now and for the future. Students found the activity meaningful, and the results from this current study show that the training outcomes concur with the competences described in validated evaluation tools of IPL activities, such as ICCAS, i.e. communication, collaboration, roles/responsibilities, person/family-centred framework, and teamwork. Compared to the ICCAS terms of competencies, the novelty of our study findings were two new categories: one’s own professional identity and non-hierarchical positioning of all involved professions.

Simulated CIP meeting as an IPL activity might be considered an activity with longstanding “effects”, as students mentioned, in “later professional life”. They might also indicate that there is a long way to go to achieve interprofessional collaboration, or that the students’ focus now is to learn about their own profession. One of the students commented that “activity with CIP might be useful in a future career”.

The model in this study, i.e. using CIP meetings with authentic cases for reaching IPL goals, might be used widely in primary care. Clinical education students might request greater awareness of and support for interprofessional training, both from university and the clinical practice. Supervisors during clinical placements education do not always take initiatives for interprofessional collaboration, and thus students may develop a tendency to continue working in silos after completing their studies. Training through simulated CIP meetings might give students the impression that interprofessional collaboration is complex work, but at the same time give them a clear idea that it is a necessary way of caring for chronically ill patients. Students see their clinical education supervisors as role models and perceive that supervisors’ attitudes are very important for developing their own attitudes to IPL [17].

It has been concluded by global experts that there is a consensus among researchers in the field that the design of IPL activities and programs needs to be adapted for local contexts to reach maximum effectiveness [29]. At the same time, they highlight several needs, one of which is to ascertain a standard, frequency and time span. Another issue is to find various ways to evaluate and measure of students’ IPL knowledge, and a third need is to clarify the integration of person-centred perspectives into the IPL.

The uniqueness of simulated CIP meetings, using typical patient cases, as an IPL activity is that (a) it is a reality-based collaboration tool that it is highly accurate for primary healthcare today, in accordance with the Act on Collaboration at Discharge from Inpatient Health Care (LUS, SFS 2017:612); (b) it partly promotes a person-centred perspective in interprofessional interaction because the patient is represented in the room during the entire activity; and (c) partly, students are invited both from the healthcare professions and other professions (e.g. social workers) trained in municipal elderly care, who are usually not included in primary healthcare clinical education. Thus, the IPL activity gains an interprofessional scope extended beyond the principal boundary and has the potential to offer students authentic and meaningful challenges.

IPL is important for students in care professions performing clinical placement in primary healthcare as it counteracts hierarchical thinking and working in silos. IPL is a pedagogic contribution that enables students to learn to work with a patient-centred approach.

Bringing together students from different medical programs/professions during clinical placement for common learning activity is a logistic challenge due to clinical placement taking place at different times. The geographic distance between placements is also wide because of the large number of care units in Region Stockholm. Therefore, it is crucial to offer learning activities that are relevant and important from the clinical perspectives of all participating professions. Additionally, important aspects for experiencing learning activities as relevant is that the activities reflect real challenges in healthcare and offer knowledge with a concrete scope (sphere), authentic case. It is important that learning activities are experienced as authentic; stimulating, self-sufficient (independent) learning is one way to promote authenticity. An alternative approach is to include simulation-based training together with IPL during clinical placement. Self-sufficient (independent) learning and a sense of authenticity are promoted through simulation-based training [30].

To implement the law according to the coordination of the care of patients with multiple needs and the coordination of healthcare and community care contributions are needed. The law applies to all professions; according to the law, patients are offered a meeting with all the relevant professionals from Health and Community care. At the meeting, a care plan created with the patient’s view of their own needs must be discussed. Healthcare providers need to understand the contribution of the CIP for the unique perspectives it gives to the care plan created during the meeting. That was why Hässelby APHCC used CIP as a basis for IPL activity using authentic patient cases. Through this type of training, future health givers will be prepared to use CIP and understand the importance of interprofessional collaboration.

### Limitations

The first limitation of this study is that evaluating one’s own knowledge of the IPL activity using authentic patient cases (CIP) through the use ICCAS might influence students’ perceptions of the IPL activity. However, ICCAS is well-validated and developed for use in learning and teaching situations within clinical placements in primary healthcare [20]. A second limitation is that we were not able to show the effectiveness of simulated CIP meetings measured by ICCAS before and after the sessions. However, it was not the purpose of this study. The character and method of content analysis was to identify in-depth the themes in students’ written free texts regarding their experiences and perspectives. The results of our study can be used for future exploration of the evaluation of CIP as an IPL activity in surveys, and other quantitative methods of evaluation of students’ clinical placements. The third limitation is that only students placed at the Hässelby APHCC and its network of healthcare centres for one year during clinical placements were invited and participated in the learning activities. However, both the limited time of clinical adjunct teachers and the logistics of clinical placements within primary care did not allow all students to be invited, as every medical program had, and still has, separate schedules for placements in different time periods.

### Strengths

This study has several strengths. Firstly, students evaluated the use of CIP as a real-life case, relevant for IPL. Secondly, having ICCAS as a basis for discussion during IPL activity is valid and helpful. Thirdly, students reported having gained new knowledge regarding others, and their own professional identity in interaction with other professions. The high number of participants in the IPL activity gives a basis for larger quantitative research studies on CIP as a highly relevant method within IPL for preparing students for future work with patients. As CIP is a law and is broadly used in primary healthcare in Sweden, we suggest that students’ discussions based on authentic cases might also help healthcare centres and raise the importance of teamwork and collaboration between different professions in primary healthcare. As clinical placements in healthcare vary (shorter for some professions and longer for others), finding situations, and establishing the logistics for common IPL activities within clinics and units would seem to be of great importance. Reaching the IPL goal will be more effective if more clinical and practical methods and tools are used. We strongly suggest that simulated CIP is one such tool.

### Implications

Using a simulated CIP-seminar with an authentic patient case as a learning activity within primary healthcare settings for IPL training to reach IPL goals might be tested further, specifically in primary care. Interprofessional collaboration that puts the patient at the centre highlights how important it is that students are prepared for patient-centred care in their future professions. Several studies emphasize that the delivery of person-centred care and improved patient outcomes, as well as better healthcare systems, relies on interprofessional collaboration as a key strategy [31–33]. Primary care university units in Region Stockholm, both at local and national levels, could effectively share study results and experiences of simulated CIP with primary healthcare networks.

## Conclusions

Simulated CIP meetings were perceived as a relevant IPL activity in the clinical training of students. Students found the activity meaningful, and the current study’s results concur with the dimensions described in validated IPL activity evaluation tools, such as the ICCAS self-assessment of IPL competencies. The novel findings in this study were that students were enabled to create new knowledge about their own professional identity and the importance of non-hierarchical teamwork. It is important to include authentic cases as a strategy in IPL activities within professional training, and to be aware that it effectively promotes professional identity building.

## Electronic Supplementary Material

Below is the link to the electronic supplementary material.

Supplementary Material 1

## Data Availability

The data (interviews) analyzed in the current study are available from the corresponding author upon reasonable request.

## References

[1] Nisbet G, O´keef M, Henderson A. Twelve tips for structuring student placements to achieve interprofessional learning outcomes. MedEdPublish. 2016. 10.15694/mep.2016.000109.

[2] O'Keefe M, Henderson A, Chick R. Defining a set of common interprofessional learning competencies for health profession students. Med Teach. 2017;39(5):463–8. 28332419 10.1080/0142159X.2017.1300246

[3] Silén C, Bolander Laksov K. To create pedagogic meetings in medicine and care. Att skapa pedagogiska möten i medicin och vård (1. uppl.). Studentlitteratur, 2013.

[4] INACSL. INACSL standards of best practice: simulation facilitation. Clinical simulation in nursing 2016;12:S16-20, 5p. Supplement: S. 10.1016/j.ecns.2016.09.007.

[5] Ulin K, Olsson L-E, Wolf A, Ekman I. Person-centred care – an approach that improves the discharge process. Eur J Cardiovasc Nursing: J Working Group Cardiovasc Nurs Eur Soc Cardiol. 2016;15(3):e19–26. 10.1177/1474515115569945. 10.1177/1474515115569945 25648848

[6] Pirhonen L, Bolin K, Olofsson EH, Fors A, Ekman I, Swedberg K, et al. Person-centred care in patients with acute coronary syndrome: cost-effectiveness analysis alongside a randomised controlled trial. Pharmacoecon Open. 2019;3(4):495–504. 10.1007/s41669-019-0126-3 PMC6861393 30825129

[7] Fors A, Blanck E, Ali L, Ekberg-Jansson A, Fu M, Lindstrom Kjellberg I, et al. Effects of a person-centred telephone-support in patients with chronic obstructive pulmonary disease and/or chronic heart failure - a randomized controlled trial. PLoS ONE. 2018;13(8):e0203031. 10.1371/journal.pone.0203031 PMC6118377 30169539

[8] Fox A, Reeves S. Interprofessional collaborative patient-centred care: a critical exploration of two related discourses. J Interprof Care. 2015;29(2):113–8. 10.3109/13561820.2014.954284 25180630

[9] Investigations. SP. Good and cose care - Care in collaboration. Good och nära vård-Vård i Samverkan. SOU 9:29:2019. Available from: https://www.regeringense/49c319/contentassets/3fcc1ab1b26b47bb9580c94c63456b1d/god-och-nara-vard-sou-2019-29pdf.

[10] Haber J, Hartnett E, Allen K, Hallas D, Dorsen C, Lange-Kessler J, et al. Putting the mouth back in the head: HEENT to HEENOT. Am J Public Health. 2015;105(3):437–41. 10.2105/AJPH.2014.302495 PMC4330841 25602900

[11] Hartnett E, Haber J, Catapano P, Dougherty N, Moursi AM, Kashani R, et al. The impact of an interprofessional pediatric oral health clerkship on advancing interprofessional education outcomes. J Dent Educ. 2019;83(8):878–86. 10.21815/JDE.019.088 31010889

[12] Janotha BL, Tamari K, Evangelidis-Sakellson V. Dental and nurse practitioner student attitudes about collaboration before and after interprofessional clinical experiences. J Dent Educ. 2019;83(6):638–44. 10.21815/JDE.019.073 30910927

[13] Singer Z, Fung K, Lillie E, McLeod J, Scott G, You P, et al. Interprofessional education day - an evaluation of an introductory experience for first-year students. J Interprof Care. 2018;32(3):399–402. 10.1080/13561820.2018.1433641 29424623

[14] El-Awaisi A, Awaisu A, Jaam M, Saffouh El Hajj M, Verjee MA. Does the delivery of interprofessional education have an effect on stereotypical views of healthcare students in Qatar? J Interprof Care. 2020;34(1):44–9. 10.1080/13561820.2019.1612863 31064272

[15] Smith LM, Keiser M, Turkelson C, Yorke AM, Sachs B, Berg K. Simulated interprofessional education discharge planning meeting to improve skills necessary for effective interprofessional practice. Prof Case Manage. 2018;23(2):75–83. 10.1097/NCM.0000000000000250 29381672

[16] Minnesota University. Standardized patient program. Engaging patients and communities in health sciences education to improve healthcare. 2019. Available from: https://www.simulation.umn.edu/standardized-patient-program.

[17] Tran C, Kaila P, Salminen H. Conditions for interprofessional education for students in primary healthcare: a qualitative study. BMC Med Educ. 2018;18(1):122. 10.1186/s12909-018-1245-8 PMC5987484 29866079

[18] Froberg M, Leanderson C, Flackman B, Hedman-Lagerlof E, Bjorklund K, Nilsson GH, et al. Experiences of a student-run clinic in primary care: a mixed-method study with students, patients and supervisors. Scand J Prim Health Care. 2018;36(1):36–46. 10.1080/02813432.2018.1426143 PMC5901439 29368978

[19] WHO. Interprofessional collaborative practice in primary health care: nursing and midwifery perspectives. 2013.

[20] Schmitz CC, Radosevich DM, Jardine P, MacDonald CJ, Trumpower D, Archibald D. The interprofessional collaborative competency attainment survey (ICCAS): a replication validation study. J Interprof Care. 2017;31(1):28–34. 10.1080/13561820.2016.1233096 27849422

[21] Riksdagen. 2 kap. 7 § Socialtjänstlagen 2001:453 och 16 kap. 4 § Hälso- och sjukvårdslagen 2017:30.

[22] Stockholm Region. Vårdgivarguiden. 2018.

[23] Archibald D, Trumpower D, MacDonald CJ. Validation of the interprofessional collaborative competency attainment survey (ICCAS). J Interprof Care. 2014;28(6):553–8. 10.3109/13561820.2014.917407 24828620

[24] Kolb D. Experiential learning - experience as a source of learning and development. Upper Saddle River, NJ: Prentice Hall; 1984.

[25] Clark PG. What would a theory of interprofessional education look like? Some suggestions for developing a theoretical framework for teamwork training 1. J Interprof Care. 2006;20(6):577–89. 17095437 10.1080/13561820600916717

[26] D’Eon M. A blueprint for interprofessional learning. J Interprof Care. 2005;19(Suppl 1):49–59. 16096145 10.1080/13561820512331350227

[27] Howard G, Dailey P. Response-shift bias: a source of contamination of self-report measures. J Appl Psychol. 1979;64(2):144–50. 10.1037/0021-9010642144.

[28] Graneheim UH, Lundman B. Qualitative content analysis in nursing research: concepts, procedures and measures to achieve trustworthiness. Nurse Educ Today. 2004;24(2):105–12. 10.1016/j.nedt.2003.10.001 14769454

[29] Rogers GD, Thistlethwaite JE, Anderson ES, Abrandt Dahlgren M, Grymonpre RE, Moran M, et al. International consensus statement on the assessment of interprofessional learning outcomes. Med Teach. 2017;39(4):347–59. 10.1080/0142159X.2017.1270441 28024436

[30] van, der Gijp A, Ravesloot CJ, Tipker CA, de Crom K, Rutgers DR, van der Schaaf MF, et al. Increasing authenticity of simulation-based assessment in diagnostic radiology. Simul Healthc. 2017;12(6):377–84. 29194106 10.1097/SIH.0000000000000278

[31] Bachynsky N. Implications for policy: the triple aim, quadruple aim, and interprofessional collaboration. Nurs Forum. 2020;55(1):54–64. 10.1111/nuf.12382 31432533

[32] Bosch B, Mansell H. Interprofessional collaboration in health care: lessons to be learned from competitive sports. Can Pharm J (Ott). 2015;148(4):176–9. 10.1177/1715163515588106 PMC4530359 26448769

[33] Reeves S, Fletcher S, Barr H, Birch I, Boet S, Davies N, et al. A BEME systematic review of the effects of interprofessional education: BEME guide 39. Med Teach. 2016;38(7):656–68. 10.3109/0142159X.2016.1173663 27146438

[34] Ashcroft R. The declaration of Helsinki. The Oxford textbook of clinical research ethics. Oxford: Oxford University. 2008:141–8.

[35] C-IPLS. Stockholm’s regional knowledge center for interprofessional learning and collaboration and division of teaching and learning at Karolinska Institutet. Handbook for Interprofessional learning and collaboration: https://www.sodersjukhuset.se/globalassets/dokument/fouui/utbildning/handbok_interpr_larande_webb.pdf.

