# Supplementary material for: Students’ experiences of interprofessional learning by simulating the Swedish concept of coordinated individual care in primary healthcare: a qualitative analysis
Source: BMC Med Educ. 2025 Jun 28;25:858. doi: 10.1186/s12909-025-07563-3 (PMC12206358; doi:10.1186/s12909-025-07563-3)
Supplement: Supplementary file 1 — Supplementary Material 1 [file 12909_2025_7563_MOESM1_ESM.docx]

Supplementary Table 1. Study population, n=87 students.

| Dates and number of participants by learning activity (n) | Medical programs | Number of units where students were placed |
| --- | --- | --- |
| **2018** |  |  |
| 09 September − n=11 |  |  |
|  | nurse (n=3)  social worker (n=2)  medical doctor (n=4)  dietician (n=1)  medical secretary (n=1) | 4 |
| 04 0ctober − n=4 |  |  |
|  | nurse (n=1)  social worker (n=1)  medical doctor (n=1)  assistant nurse (n=1) | 2 |
| 22 November − n=5 |  |  |
|  | nurse (n=2)  social worker (n=1) physiotherapist (n=2) | 3 |
| 29 November − n=10 |  |  |
|  | nurse (n=2)  social worker (n=1)  medical doctor (n=1)  occupational therapist (n=2) district nurse (n=1) chiropractor (n=1)  naprapathy (n=1) | 4 |
|  |  |  |
| **2019** |  |  |
| 07 February − n=5 |  |  |
|  | nurse (n=2)  medical doctors (n=3) | 3 |
| 28 March − n=9 |  |  |
|  | nurse (n=4)  social worker (n=2)  occupational therapist (n=1) physiotherapist (n=2) | 6 |
| 02 May − n=6 |  |  |
|  | nurse (n=2)  social worker (n=1)  medical doctor (n=1)  medical secretary (n=1) occupational therapist (n=1) | 3 |
| 16 May − n=6 |  |  |
|  | nurse (n=1)  district nurse (n=1)  medical doctor (n=1)  assistant nurse (n=1) physiotherapist (n=2) | 13 |
| 19 September − n=7 |  |  |
|  | nurse (n=3)  social worker (n=1)  medical doctor (n=2)  medical secretary (n=1) | 2 |
| 17 October − n=10 |  |  |
|  | nurse (n=4)  district nurse (n=2) | 11 |
|  | social worker (n=4)  medical doctor (n=1)  medical secretary (n=1) occupational therapist (n=2) |  |
| 28 November − n=8 |  |  |
|  | nurse (n=1)  district nurse (n=2)  physiotherapist (n=2)  paediatric nurse (n=2) | 5 |
| 12 December − n=6 |  |  |
|  | nurse (n=1)  district nurse (n=2)  medical doctor (n=2) physiotherapist (n=1) | 4 |

Supplementary Table 2. Results of content analysis with theme, categories, and subcategories.

| **THEME**: Interprofessional collaboration without hierarchy with patient in center | |
| --- | --- |
| **Categories** | **Subcategories** |
| Patient in center | Collaboration with the patient  To do the best for the patient  To represent the patient  A holistic view of the patient |
| One’s own professional identity | One’s own experiences  Building one’s own identity  Building one’s own profession  Professional responsibility |
| Professional identity of others | New knowledge of other professions  Acceptance of others  Understanding roles and responsibilities |
| Teamwork and collaboration | Collaboration  Organizational responsibility  Communication with each other  Help each other |
| Non-hierarchical positioning | Everyone is needed  Everyone needs to contribute  Everyone is equal |
